# Refractory systemic onset juvenile idiopathic arthritis: current challenges and future perspectives

**DOI:** 10.1080/07853890.2022.2095431

**Published:** 2022-07-04

**Authors:** William G. Ambler, Kabita Nanda, Karen Brandt Onel, Susan Shenoi

**Affiliations:** a Division of Pediatric Rheumatology, Hospital for Special Surgery, New York, NY, USA; b Department of Pediatrics, Weill Cornell Medical College, New York, NY, USA; c Department of Pediatrics, Division of Rheumatology, University of Washington School of Medicine & Seattle Children’s Hospital, Seattle, WA, USA

**Keywords:** Systemic onset juvenile idiopathic arthritis, refractory disease

## Abstract

Systemic juvenile idiopathic arthritis (SJIA) is a rare disease with distinct features not seen in other categories of juvenile idiopathic arthritis. In recent years, advances in the understanding of disease immunopathogenesis have led to improved targeted therapies with significant improvement in patient outcomes. Despite these advances, there remain subsets of SJIA with refractory disease and severe disease-associated complications. This review highlights existing options for treatment of refractory SJIA and explores potential future therapeutics for refractory disease.

**Key Points:**
- Despite targeted Interleukin IL-1 and IL-6 inhibitors a subset of SJIA remains refractory to therapy. About 1 in 7 SJIA patients will be refractory to targeted IL-1 or IL-6 therapy.
- There is no current agreed upon definition for refractory SJIA and we propose in this review that refractory SJIA is presence of active systemic or arthritic features despite treatment with anti-IL-1 or anti-IL-6 therapy or disease requiring glucocorticoids for control beyond 6 months.
- SJIA disease associated complications include presence of associated macrophage activation syndrome (MAS), interstitial lung disease (ILD) or amyloidosis and management of each differs.
- Refractory SJIA treatment options currently include additional conventional synthetic disease modifying anti-rheumatic drugs (csDMARDS), biologic (bDMARDS), combination biologic therapy, targeted synthetic (tsDMARDS) or other immunomodulatory therapies.

## Background

The International League of Associations for Rheumatology (ILAR) classifies systemic onset juvenile idiopathic arthritis (SJIA) as occurring in children under age 16 with fever for at least 2 weeks, of which there must be 3 consecutive days of quotidian fever, arthritis in one or more joints and with at least one of the following: evanescent erythematous rash, generalised lymphadenopathy, hepatomegaly, splenomegaly, or serositis [1]. Though most forms of JIA are conceptualised as autoimmune diseases, SJIA uniquely has many features of autoinflammatory disease. This has led to a paradigm shift in the treatment of patients, specifically due to the recognition of two critical cytokines in disease pathophysiology. Successful trials using Interleukin (IL)-1 and IL-6 inhibition have led to their widespread use and have dramatically improved patient outcomes [2,3]. IL-1 or IL-6 inhibitors are now recommended as first line therapy [4–7]. Multiple long term outcome studies demonstrate that most patients (60–80%) can achieve disease remission or minimal disease activity [8–12]. Effective treatment of patients earlier in the disease course may change long-term outcomes with fewer patients progressing to chronic synovitis [13]. SJIA disease course varies with approximately 40% having a monocyclic disease course, 10% with a polycyclic course, and 50% with a chronic course [14–16]. Patients with a monocyclic course have a brief duration of active disease (usually less than 6 months) and have excellent outcomes. Prior to the advent of targeted biologic therapies, patients with a chronic disease course often went on to have severe, erosive, polyarticular arthritis often requiring long term glucocorticoids for arthritis control resulting in associated glucocorticoid toxicity [15,17]. Even in the post targeted biologic era refractory disease in 20% with ongoing systemic symptoms and/or synovitis poses a significant challenge. Additionally, association of SJIA with a rare interstitial lung disease (ILD) in certain high-risk patients can pose significant treatment challenges with morbidity and mortality. Macrophage activation syndrome (MAS), a life-threatening cytokine storm, is also a complication in a subset of patients. This review proposes a definition for refractory SJIA and reviews existing options for management of refractory SJIA. We also discuss SJIA associated complications including MAS, ILD and amyloidosis and discuss their management.

## Pathophysiology

Understanding the immunologic mechanisms of SJIA has been and will continue to be critical to the development of effective treatments. The innate immune system is heavily implicated in disease pathophysiology. Myeloid cells are increased in number and activation status in the peripheral blood of SJIA [18,19]. Similarly, innate cytokines and alarmins are elevated in patients’ sera [20]. Pascual *et al.* demonstrated that SJIA sera can increase monocyte transcription of inflammatory cytokines, including IL-1β [21]. Activated monocytes from patients with SJIA release significantly more IL-1β than controls. These findings led to the successful treatment of many SJIA patients with anakinra, an IL-1 receptor antagonist. Subsequently, IL-1 blockade with canakinumab has been further studied and has gained United States Food and Drug Administration (US FDA) approval for the treatment of SJIA. Approximately 60% of patients can achieve remission on IL-1 antagonism as first line therapy [13,22]. IL-6 has also been implicated in SJIA, with increased IL-6 and IL-10 gene expression in SJIA monocytes and B cells compared to controls [23]. IL-6 blockade has similarly been effective, and tocilizumab is US FDA approved for SJIA treatment.

Though innate immune pathways are clearly important, recent evidence points to a role of the adaptive immune system as well. Ombrello *et al.* conducted GWAS on a large cohort of SJIA and demonstrated that MHC class II alleles portend the largest risk for SJIA development [24,25]. Further, the gene architecture of SJIA is divergent from other JIA categories [25]. These findings are supported by studies demonstrating the importance of T cells in chronic synovitis [26,27]. By analysing patients’ T cells, Henderson *et al.* demonstrated that different T cell subsets were present in different disease stages [27]. In the early inflammatory stage of disease, genuine IL-17 producing regulatory T cells were expanded. In patients that developed chronic synovitis, IL-17 producing effector CD4 cells were expanded. Patients who achieved early remission with IL-1 blockade failed to expand IL-17 effector CD4 T cells. This provides evidence to the theory of a “window of opportunity,” where the early inflammatory milieu might favour an adaptive immune response leading to chronic synovitis [28]. Further, the authors hypothesise that medications targeting IL-17 (i.e. secukinumab) may be a logical treatment option for refractory synovitis [27].

MAS develops in 10–30% of patients with SJIA [29,30]. The reader is referred to several in depth reviews on this topic [29,31]. The “cytokine storm” of MAS is driven by activated macrophages and cytotoxic T cells [32]. Key findings have demonstrated the importance of IL-18 and interferon gamma (IFN-γ) in MAS. IL-18 is an inflammasome generated cytokine which induces IFN-γ release from T cells [33]. IL-18 is elevated in most patients with SJIA, however IL-18 is highest in those who develop MAS and rises further in active MAS [34]. Notably, IL-18 distinguishes MAS from primary hemophagocytic lymphohistiocytosis (HLH), in which a similar cytokine storm occurs usually in the setting of mutations in cytotoxic T cell killing [35]. IFN-γ is a common downstream cytokine released in both MAS and HLH. IFN-γ is required for HLH pathophysiology and blocking IFN-γ with emapalumab is approved for the treatment of primary HLH [36,37].

## Refractory SJIA

For the purpose of this review refractory SJIA is defined as failure to respond to IL-1 and IL-6 inhibitors or need for ongoing treatment with long term glucocorticoids (beyond 6 months) with persistence of systemic and/or arthritic features (Table 1). If a patient does have an adequate response to one of the previously mentioned cytokine inhibitors (IL-1 or IL-6 inhibitor) the other should be used. Refractory SJIA should only be determined after inadequate responses of both IL1 and IL6 blockade (though not necessarily simultaneous use). In the current treatment era with wide availability of effective medications with different targets, inability to taper glucocorticoids should be considered refractory disease due to long term toxicity, regardless of disease activity [7,38**]**. Another recent review proposes definitions for different subsets of refractory patients (refractory arthritis, refractory MAS, and ILD) [39]. These could be meaningful partitions of refractory subtypes given the likelihood of different immune drivers in patients with these different refractory phenotypes. For this review we separate out refractory SJIA (as defined above) and SJIA associated complications (including MAS, SJIA associated ILD and amyloidosis). A universally accepted definition will be helpful in order to design future outcome and treatment studies.

**Table 1.** Proposed definition of Refractory SJIA.

Condition:	Definition:
Refractory SJIA	Failure to respond to IL-1 and/or IL-6 inhibitors or need for ongoing treatment with long term glucocorticoids (beyond 6 months) with persistence of systemic and/or arthritic features
SJIA associated complications	MAS SJIA ILD SJIA amyloidosis

In the SJIA literature there exist two different time spans: the pre-biologic era, defined as the period prior to availability of anti-IL-1 and anti-IL-6 agents, and the post- biologic era. Two broad groups are used to identify refractory disease: 1) Lack of efficacy or clinical response including no response to medication, and incomplete response to medications (pertaining to either the systemic or arthritic features of SJIA); 2) Severe adverse reactions or side effects to medication necessitating discontinuation or change of therapy. Also, biologics are still not widely available in all countries. The IL-1 inhibitor canakinumab was approved by the US FDA in 2013 for the treatment of SJIA in children over 2 years of age [3]. The other anti-IL-1 agents, while not US FDA approved, have been used off label in SJIA, including anakinra [40] and rilonacept [41]. Rilonacept is a fusion protein which binds to IL-1 (IL-1 trap) and has been shown to be effective in a phase II trial with 74% of patients meeting the primary outcome of an ACR30 response at 4 weeks [41]. Ultimately the manufacturers did not choose to seek US FDA approval for SJIA and it thus remains largely unavailable. Tocilizumab was US FDA approved for SJIA in children ≥ 2 years of age in 2011 [2]. In the canakinumab trial, the primary end point was met with 83% of patients achieving an ACR30 response at 15 days (compared to 10% in placebo arm). At the end of the withdrawal phase 82% achieved an ACR70 response and 62% had clinical inactive disease (CID). In the tocilizumab trials, 85% of patients achieved the primary outcome of an ACR 30 response at 12 weeks (compared to 24% in placebo arm) and 59% achieved an ACR90 response at the end of the open label extension at 52 weeks. The ACR30 is a validated mark of minimal improvement to assess efficacy for drug approval [42]. Clinically, however, an ACR30 response is a minimal response, and the expectation is for most patients is to achieve inactive disease or low disease activity. Despite these dramatic responses, there were still approximately 15–20% of patients with no response to single drug biologic therapy and approximately 40% who continued to have active disease while on single drug biologic therapy. It should be noted that most patients in both trials had long-standing disease. Real world data in the biologic era demonstrates that most patients can achieve inactive disease at long term follow up. In a Canadian inception cohort (ReACCh-Out), 70% of SJIA achieved CID by 2 years and 85% by 5 years [10]. Similar findings were seen in other cohorts [8,43]. A long term follow up (18 year follow up) study in a Nordic cohort showed 3/13 (23%) patients continued to have active disease [11]. This study is somewhat limited by the few patients with SJIA followed. Even in the post-biologic era, approximately 1 in 7 children will continue to have long-term disease activity.

## Treatment options for refractory SJIA

### Conventional synthetic (cs) DMARDs

While methotrexate is often used as adjunctive treatment for SJIA, other csDMARDs are used with less frequency. While many of these medications are currently used infrequently, it can be reasonable to utilise them in certain situations particularly for refractory arthritic features of SJIA (Table 2). Thalidomide, an immunomodulatory drug has shown benefit in refractory SJIA. While the exact mechanism of action is not entirely understood, thalidomide decreases production of multiple inflammatory cytokines including **Tumour necrosis factor alpha (**TNF-α**)**, IL-1, and IL-6 [68]. Three small case series and one small trial provide some anecdotal evidence for its use in refractory cases [44–47]. Patients in all three-case series had severe systemic and arthritic disease, refractory to a variety of different medications, and all required high doses of glucocorticoids. After treatment with thalidomide, patients were able to achieve either inactive disease or low disease activity while lowering or discontinuing glucocorticoids. The single trial assessed the utility of thalidomide in refractory disease in 13 patients from 4 different centres. Eleven of the 13 patients had improvements in erythrocyte sedimentation rate, anaemia, at least 50% improvement in disease score, and were able to decrease glucocorticoid usage [47]. While thalidomide is a well-known teratogen and can cause neuropathy, it can still be considered, particularly in resource limited settings [68]. Lenalidomide, a thalidomide analogue with significantly less neurotoxicity, may be another option as well. Though there are currently no published reports on efficacy or safety, this author and others have used it successfully in refractory SJIA [16].

**Table 2.** Medications used in treatment of refractory SJIA.

Medication used for refractory SJIA	Mechanism of action	Relevant literature	Comments
csDMARDs			
Thalidomide	Decreases TNF-α and IL-6 secretion, decreases angiogenesis	[44–47]	Exact mechanism of action is not well described
Tacrolimus	Inhibits T cell activation and proliferation *via* inhibiting calcineurin	[48,49]	Volcosporin is newer calcineurin inhibitor with more stable pharmacokinetics
Cyclophosphamide	Alkylating agent, particularly effective at eliminating reactive lymphocytes	[50,51]	
Cyclosporine	Inhibits T cell activation and proliferation *via* inhibiting calcineurin	[38,52,53]	Also effective add on therapy for MAS and has been used for SJIA ILD
bDMARDs			
Rituximab	Elimination of non-stem B cells	[54–56]	May be effective directly or indirectly through B cell antigen presentation to T cells
TNF-α inhibitors	Inhibits TNF-α signalling	[57,58]	Pleiotropic pro-inflammatory cytokine
Abatacept	Inhibits T cell co-stimulation by blocking costimulatory molecules CD80/86 on APCs	[59,60]	
Tadekinig Alfa	Blocks IL-18 signalling by binding unbound “free” IL-18	[34,61]	
tsDMARDs			
Jakinib	Inhibits signalling through JAK/STAT pathway, inhibiting multiple cytokines	[62,63]	Different jakinibs have reported differing effect on different JAK/STAT pathways *via in vitro* studies [64] Also may be effective in MAS and SJIA ILD
Medications used for MAS			
Emapalumab	Inhibits IFN-γ signalling *via* direct binding	[65]	Dose titrated bases on clinical effectiveness. Can measure effectiveness of blockade by measurement of CXCL9.
Anti-thymocyte globulin	Polyclonal antibodies that eliminate T cells	[66]	
Etoposide	Topoisomerase II inhibitor	[67]	Effective at eliminating activated and dividing T cells. Effective at lower doses then in the HLH 94 protocol
Medications used for ILD			
MMF	Inhibits inosine monophosphate dehydrogenase leading to decreased lymphocyte activation and proliferation	[38]	Effective in slowing lung fibrosis in scleroderma
Cyclosporine	See above		
Jakinibs	See above		

APC: Antigen presenting cell, JAK: Janus Kinase, STAT: Signal Transducer and Activator of Transcription; csDMARD: Conventional Synthetic DMARD; bDMARD: Biologic DMARD; tsDMARD: targeted synthetic DMARD; ILD: Interstitial Lung Disease; HLH: Hemophagocytic Lymphohistiocytosis.

Calcineurin inhibitors, particularly cyclosporine, have been used for decades in the treatment of SJIA. Cyclosporine is particularly useful in the treatment of MAS, in which T cell secreted IFN-γ is critical [69]. Cyclosporine also has use in systemic manifestations of SJIA, however is less effective for arthritis [52,53,70]. In a retrospective study from an area where IL-1 and IL-6 inhibitors are prohibitively expensive, cyclosporine led to significant reduction in systemic symptoms and glucocorticoid dosage [53]. Of 13 patients with steroid dependent and refractory SJIA, all had resolution of systemic symptoms within one month of initiation of cyclosporine along with normalisation of serum markers of inflammation. Glucocorticoid dose was able to be decreased by an average 50% at a median of about 2 months. Tacrolimus has not been used as frequently, but in case reports has demonstrated similar efficacy [48,49]. In one recent single centre retrospective case series from Shanghai, of 6 patients with SJIA treated with tacrolimus, all had improvements in laboratory parameters and were able to lower glucocorticoids [48]. Voclosporin a newer agent has recently gained approval for adult lupus and potentially could be another therapeutic option in the future.

Cyclophosphamide (CYC), an alkylating agent with a long history of use in multiple rheumatic diseases, has demonstrated efficacy in the treatment of refractory SJIA [50,51]. In a small trial prior to the biologic era, 4 patients with severe refractory disease had positive responses to intravenous (IV) CYC given monthly [50]. All patients were ablet to discontinue glucocorticoids and 3 achieved inactive disease. In another small open label trial of refractory SJIA (pre-biologic era) 18 patients with SJIA had significant improvement in both systemic and articular symptoms after started on IV CYC at 400 mg/m^2^ every 3 months [51]. CYC is broadly immunosuppressive and has toxic effects on myeloid cells and well as lymphocytes, thus its efficacy in SJIA is not surprising [71]. However, CYC has many side effects including increased risk of malignancy and the risk of infertility limiting its usefulness in the long term. The role of CYC in the biologic era is unclear but could be considered in very select cases.

### Biologic (b) DMARDS

TNF-α inhibitors have been tried in refractory SJIA [57]. In general TNF-α inhibitors are less effective for the systemic features of the disease but may help with arthritic features of SJIA [57]. Kimura *et al.* highlight this suboptimal response in their paper that surveyed 82 SJIA patients on etanercept. Response was poor in 45%, 33% discontinued etanercept due to lack of response or flare and 2.4% developed MAS while on etanercept [58].

Rituximab, a monoclonal antibody that depletes B cells, has shown some efficacy. Forty-four patients with refractory SJIA (prior to the availability of IL-1 or IL-6 blockade) were treated with rituximab in a prospective, open label, uncontrolled trial [54]. Patients were treated every 24 weeks if there was ongoing disease activity. After 24 weeks, 98% achieved an ACR30 and 25% of patients were able to achieve remission. All but 2 patients had resolution of fever by 24 weeks. In 25 patients that had continued to be followed for 96 months, 24 achieved remission. There are also two case reports that describe significant improvement in both systemic and arthritic features in refractory cases [55,56]. While the role of B cells in the pathogenesis of SJIA is unclear there is some evidence that depleting them can be of benefit. While there are no case reports, we postulate that obinutuzumab another B cell depleting agent might be similarly beneficial in refractory SJIA.

IL-18 is another promising target as IL-18 is elevated in SJIA and correlates with the risk of developing MAS. IL-18 binds to IL-18 receptor protein and it is the unbound fraction of IL-18 which actively signals [72]. Tadekinig alfa is a recombinant IL-18 receptor protein which functionally inhibits IL-18 signalling. A phase II trial showed benefit in patients with Adult Onset Stills Disease (AOSD) [61], which could potentially translate to patients with SJIA given that AOSD is essentially identical to SJIA both in phenotype and pathophysiology [73,74]. There is single case report showing benefit of IL-18 inhibition in a child with refractory SJIA [75]. In this report IL-18 inhibition was given to 6-year-old with SJIA with recurrent MAS refractory to chronic high doses of steroids as well as numerous bDMARD and csDMARDs including IL-1 and IL-6 inhibition. After starting IL-18 inhibition the patient improved and was able to considerably lower daily steroid usage. Despite having several episodes of MAS on IL-18 therapy, the episodes were significantly less severe than prior episodes. IL-18 binding protein is currently in clinical trials for X-linked inhibitor of apoptosis inhibitor (XIAP) deficiency and NLR Family CARD Domain Containing 4 (NLRC4) gain of function mutations (NCT03113760). These diseases are characterised by elevated IL-18 and are frequently complicated by MAS/HLH. It will be interesting to see if this medication will be beneficial in patients with SJIA, particularly in children with MAS.

Other IL-6 inhibitors which likely are similarly efficacious as tocilizumab exist. Siltuximab, an IL-6 binding chimeric monoclonal antibody, was effective in a 20-year-old patient with SJIA who failed multiple therapies including IL-1 blockade and was unable to tolerate tocilizumab because of infusion reactions [76]. Sarilumab is an alternate IL-6 binding monoclonal antibody currently approved for rheumatoid arthritis that is being studied for its utility in SJIA (NCT02991469). Although these alternative medications have not been rigorously studied in SJIA, and thus currently lack specific approval, it is likely that they are effective. It is unclear if patients that fail tocilizumab might respond to a different IL-6 inhibitor and more data is needed to evaluate this possibility.

Combination biologic therapy may be beneficial in some refractory patients. One case series reports a 17 year old patient with SJIA with persistent systemic and arthritic disease on tocilizumab monotherapy and anakinra monotherapy (with concomitant methotrexate and glucocorticoids) [77]. However, when low doses of tocilizumab (2 mg/kg) and anakinra were used simultaneously, the patient achieved inactive disease. Abatacept has been used with success in combination with anakinra in a case series [59]. Abatacept, a soluble cytotoxic T-lymphocyte associated protein 4 fused the Fc portion of immunoglobulin (CTLA-4-Ig), inhibits T cell co-stimulation [60]. In this case series, 4 patients with glucocorticoid dependent disease despite use of anakinra, methotrexate, and cyclosporine had significant improvement in disease control and were able to reduce glucocorticoid dosage with the addition of abatacept Given the importance of T cells in the development of chronic synovitis in SJIA, this may be a promising option as add-on therapy. Despite the more frequent use of combination therapy, there is minimal data on the safety or efficacy of combination therapy.

### Targeted synthetic (ts) DMARDs

Janus kinase inhibitors (Jakinibs) are a promising option based on the profile of cytokine signalling pathways that are inhibited. A few such cytokines are IFN-γ and IL-6, which as previously mentioned are important in the pathophysiology of SJIA. An in depth review regarding the theoretic benefits of Jakinibs in SJIA is detailed in a recent review by Verweyen and Schulert [78]. There is currently a clinical trial using tofacitinib for treatment of children with SJIA with active systemic features (NCT03000439). While mechanistically Jakinibs are intriguing, there are only few case reports published of their use in the treatment of SJIA. One case report from China describes a 13-year-old girl with recalcitrant systemic and arthritic manifestations of SJIA despite long term glucocorticoids, methotrexate, and TNF-α inhibition [78]. The patient suffered significant glucocorticoid toxicity with vertebral compression fractures and growth retardation. Tofactinib was started due to family’s refusal of tocilizumab and unavailability of IL-1 antagonism in mainland China. The patient had complete remission by 3 months and discontinued glucocorticoids by 6 months. Though this patient may not have been refractory if given IL-1 or IL-6 inhibitors, this case report does support the potential benefit of Jakinibs in SJIA. Success using Jakinibs in AOSD, may translate to SJIA given their previously mentioned similarities [73,74]. In a case series of 14 patients from China who received tofacitinib, 7 achieved remission and the remainder were partial responders (using the modified Pouchot’s systemic score) [62]. Only 2 patients had received tocilizumab prior to receiving tofacitinib and both were partial responders. Another case report demonstrates success with the use of ruxolitinib in a 4 year old SJIA complicated by drug reaction to anakinra and canakinumab with development of ILD [63]. After 15 months of follow up the patient had resolution of systemic symptoms, normalisation of C-reactive protein, improvement in oxygen saturation and lung imaging, and was able to lower glucocorticoid usage. Results from the tofacitinib trial for SJIA (NCT03000439) are eagerly awaited as it may expand the existing armamentarium of therapeutic options for SJIA and will likely reduce the number of refractory disease patients.

### Stem cell therapies

Bone marrow transplantation has been used for refractory disease in both the pre- and post-biologic eras. Earlier work from several groups in Europe studied T cell depleted autologous haematopoietic stem cell transplantation (HSCT) as a strategy to induce remission [79,80]. The Dutch group (Brinkman *et al.*) used this strategy in 18 patients with refractory SJIA [79]. While 6 achieved remission (per Wallace criteria) and 5 more had a partial response (>ACR 50%) there was significant morbidity and mortality. Two patients died from MAS early post-transplant, which had been reported in prior reports. An additional 2 patients died after one year from viral infection. A group from the United Kingdom had a case series of 7 patients that underwent HSCT and 4 achieved long lasting remission, 2 had disease relapse, and 1 died from viral infection [80]. While autologous HSCT can result in long lasting remission, a large proportion have disease recurrence and there is significant morbidity and mortality with rates of approximately 15–22% [79,80]. Since MAS accounts for a large portion of the mortality post-transplant it is critical to attain minimal disease activity prior to transplant in order to improve transplant related outcomes [81].

Allogeneic HSCT has a higher chance of lifelong cure, however there is also risk of graft versus host disease (∼20%) [82]. Silva *et al.* published the results of performing allogenic HSCT on 16 patients with refractory JIA (11 of whom had SJIA). Many of these patients were refractory to IL-1 and IL-6 blockade. These patients received reduced intensity conditioning with fludarabine based regimens and alemtuzumab. Of the 11 SJIA patients reported, 1 died due to pulmonary haemorrhage, 1 had disease recurrence necessitating reintroduction of medications, and 9 achieved clinical remission. Post-transplant complications included graft versus host disease in 5 patients and viral infections (several severe) in most patients. Early MAS was not observed as it was in autologous HSCT [82]. Allogeneic stem cell transplantation may have a role in refractory disease; however, this procedure comes with known morbidity and mortality risk [82].

Mesenchymal stromal cell (MSC) infusions represent another possible therapeutic option for refractory SJIA cases. MSC are non-embryonic, fibroblast-like stem cells that are isolated from blood or bone marrow and have immunoregulatory properties [83]. They can inhibit T cells, B cells, natural killer (NK) cells, and dendritic cells and can activate Regulatory T cells. They also express very little major histocompatibility complex class I (MCH class I) and thus do not invoke an alloimmune reaction. Swart *et al.* did a proof-of-concept phase 1 b trial with 6 refractory JIA patients (1 with SJIA). The patient with SJIA was refractory to multiple therapies including IL-1 and IL-6 blockade. This patient was able to wean glucocorticoids, however developed MAS when attempting to discontinue tocilizumab. While clearly warranting additional study, MSC may have a future role in SJIA and other immune mediated disease.

## Management of SJIA complications

### Macrophage activation syndrome

MAS, a life-threatening manifestation of SJIA, can be difficult to treat. Treatments vary based on severity, but standard treatments include high doses of IV glucocorticoids, cyclosporine, and high doses of anakinra [31,84]. The ACR recently published guidelines for management of SJIA associated with MAS [6]. Intravenous immunoglobulin (IVIG) has been used with mixed success in several case reports and could be considered in refractory cases [85,86]. IFN-γ is a critical cytokine in HLH/MAS pathophysiology [36,87]. Emapalumab, a monoclonal antibody that binds IFN-γ, is effective and has gained approval for the treatment of refractory primary HLH [37]. Preliminary clinical trial data (NCT03311854) suggests that IFN-γ blockade is safe and effective. Nine patients with refractory MAS to high dose IV glucocorticoids that have been treated with emapalumab have been analysed thus far. Of these patients, 4 had prior treatment failure with cyclosporine and 4 with anakinra. All patients had resolution of clinical and laboratory parameters of MAS with emapalumab treatment [65]. IFN-γ blockade represents a promising new drug candidate in the armamentarium for treatment of refractory MAS in SJIA. Anti-thymocyte globulin (ATG) and etoposide, both medications effective at depleting T cells, have been used for refractory cases with success [66,67]. Lower doses of etoposide (when compared with doses per the HLH-94/2004 protocol) are likely efficacious for refractory MAS [67]. In 7 patients with refractory MAS (including 2 with SJIA), weekly etoposide at 50–100mg/m^2^ was used successfully in all patients [67]. In the acute setting there may be a role for leukocytapheresis, particularly for refractory MAS [88–90]. However, application of this is limited due to the temporary nature of action and need for central line access limiting use to tertiary care settings.

### Systemic JIA associated lung disease

In the last several decades there are increasing numbers of a subset of children with SJIA that developed a severe and often fatal lung disease with a mortality rate around 60% [91]. These patients develop a pulmonary alveolar proteinosis-like (PAP) ILD. Compared to SJIA children without ILD, those with ILD have a younger age at disease onset, more frequent MAS, atypical rashes, less prominent arthritis, and frequently develop drug reactions to biologic medications including tocilizumab, anakinra, and/or canakinumab. The exact aetiology of this complication is unclear. Saper and Ombrello *et al.* showed that there is a strong association of this phenotype with a certain HLA haplotype (HLA-DRB1*15 alleles) [92]. Schulert *et al.* demonstrated that these patients have elevated IL-18 and the IFN-γ stimulated gene CXCL9, which would be expected in a group that develops more frequent MAS [93]. Lung biopsies demonstrated T cell infiltration with upregulation of gene transcription in both IFN-γ pathways and T cell activation pathways. Based on these observations, a variety of different treatments have been used in attempt to improve the outcomes of these patients. Some clinicians discontinue biologic therapy, and in addition to high doses of glucocorticoids, utilise drugs that target both T cells and/or IFN-γ including cyclosporine, mycophenolate mofetil, and Jakinibs with reported success [38,63]. This is an area of active research to both determine the mechanistic cause of PAP-like ILD and to how to most effectively treat this group of patients.

### Amyloidosis

Secondary amyloidosis (AA amyloid), caused by the deposition of serum amyloid A (SAA), is a life-threatening complication of prolonged systemic inflammation [94]. This usually manifests as renal dysfunction and has a high mortality rate. Prior to the biologic era, amyloidosis was seen in increased frequency in SJIA. In one long term follow up study in 2002 from a group in the UK, the rate of amyloidosis in SJIA was approximately 20%. Fortunately, with modern therapies, amyloidosis is now rarely seen in SJIA and recent rates have not been reported. There are still case reports of AA amyloid, always in the context of chronically active disease [95]. Effective treatment of the underlying disease is critical to the treatment of amyloid, as reduction in systemic inflammation reduces production and deposition of SAA. Tocilizumab or other IL-6 inhibitors may be of particular benefit as IL-6 has been shown to be important in hepatic SAA production [95,96].

### Future directions

Scientific discovery has led to radical improvements in SJIA outcomes through a deepened understanding of the immunobiology and pathophysiology of disease. Despite this there are still patients that do not respond to contemporary first line treatment with IL-1 or IL-6 inhibition. Patients with ILD also pose significant new challenges. Additional drugs will likely become available for use in our armamentarium to treat SJIA with further scientific advances. Jakinibs, IL-18 receptor blockade, and IFN-γ receptor blockade are agents that will hopefully prove to be safe and effective for patients with SJIA. There may be opportunities for personalised medicine based on individualised patient phenotype, dysregulated immune pathways, and genetics [97,98]. Translational and clinical studies will hopefully continue to lead to effective treatment discoveries to reduce the number of patients with refractory disease and to improve patient outcomes.

## Author contributions

WA contributed to the literature review, the design of work, and wrote the original manuscript. KN contributed to literature review and critically revised the article. KO contributed to literature review, assisted in the design of the work, and critically revised the article. SS supervised the literature review, conceptualised, and designed the work, and critically revised the article. All authors approved the final manuscript approval.

## Data Availability

Data sharing is not applicable to this article as no new data were created or analysed in this study.
